# Development of an automated REM sleep deprivation device for mice in neuroscience research

**DOI:** 10.1016/j.ohx.2026.e00797

**Published:** 2026-05-23

**Authors:** Ruilin Yang, Wyatt D. Morse, Peng Zhong

**Affiliations:** Department of Neurological Sciences, University of Nebraska Medical Center, Omaha, NE 68106, USA

**Keywords:** REM sleep deprivation, Closed-loop control, EEG-based detection, Open-source hardware, Home-cage sleep deprivation

## Abstract

Rapid eye movement (REM) sleep deprivation paradigms are widely used to investigate the functional role of REM sleep, yet conventional methods often disrupt natural housing conditions and introduce stress-related confounds. Here, we present an open-source, home-cage–compatible system for automated REM sleep deprivation in mice that integrates physiological monitoring with real-time closed-loop motor control. Electroencephalography (EEG) and electromyography (EMG) signals are acquired using an Open Ephys acquisition board coupled to an Intan RHD-series headstage (Intan Technologies, USA). Real-time REM detection relies solely on EEG-derived spectral features, whereas offline brain-state annotation is performed using combined EEG and EMG recordings. The real-time classifier discriminates REM, wake, and non-rapid eye movement (NREM) states with accuracies of 88.6%, 80.4%, and 89.1%, respectively. Upon REM detection, the Open Ephys board outputs a Transistor–Transistor Logic (TTL) signal to a servo motor controller (Maxon ESCON 50/5), which drives a gently moving platform to selectively disrupt REM episodes. During a continuous 48 h deprivation protocol, REM sleep was robustly suppressed. At 24 h, the baseline-to-deprivation ratio for REM was approximately 15.9x, while wake and NREM ratios were approximately 0.66x and 1.46x, respectively. Sustained REM suppression remained evident at 48 h (∼7.9x), demonstrating stable closed-loop performance. All hardware design files and source code are publicly available.

## Highlights

• Home-cage–compatible closed-loop REM manipulation in freely moving mice (N = 8).
• Real-time EEG-only classification achieves 88.6% REM, 80.4% Wake, and 89.1% NREM accuracy.
• REM sleep suppressed by >90% at 24 h (15.9-fold) and sustained at 48 h (7.9-fold) with minimal off-target effects.
• Low-cost, open-source, Open Ephys–based platform for scalable neuroscience research.

## Graphical abstract

**Figure:**
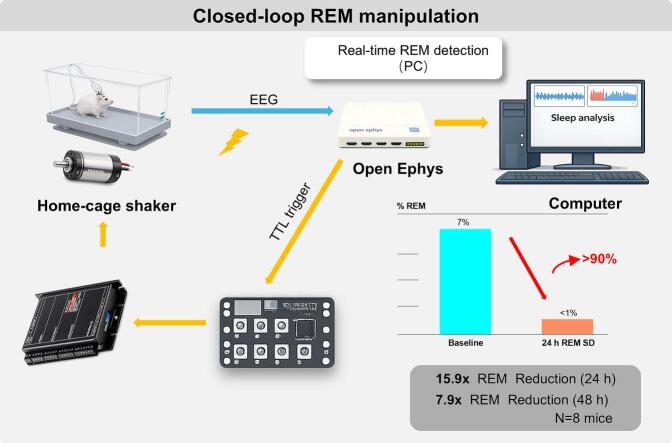

## 1 Specifications table

**Table:** 

Hardware name	An Affordable REM Sleep Deprivation Device
Subject area	Neuroscience research
Hardware type	Electrical engineering and computer scienceMechanical engineering and materials science
Closest commercial analog	No commercial analog is available
Open source license	*CC BY 4.0*
Cost of hardware	*$6290.39*
Source file repository	Open Science Framework https://doi.org/10.17605/OSF.IO/R3U5G

## 2 Hardware in context

Sleep is separated into two main types: rapid eye movement (REM) sleep and non-REM (NREM) sleep, both of which are tightly controlled by distinct neural circuits. Known as a paradoxical sleep state, REM sleep is characterized by a desynchronized electroencephalogram (EEG) similar to wakefulness, cortical activation, and low electromyogram (EMG) activity indicating muscle atonia, and is associated with vivid dreaming [1], [2], [3]. Although a seemingly unproductive behavioral state, it functions to maintain affective brain homeostasis and support emotional functioning [4], facilitate motor learning [5], and promote the formation and consolidation of memory [6], [7], [8], [9]. Although the brain mechanisms controlling REM sleep have been studied extensively [10], [11], the underlying neural circuits are only partially understood. Sleep deprivation has been widely used to identify neuronal populations controlling REM sleep and to investigate the contribution of REM sleep to memory, learning, and emotional regulation [12]. This paradigm enables controlled examination of the effects of sleep loss and recovery on neural circuits and their physiological outputs.

Traditional approaches to REM sleep deprivation (RSD), including the small-platforms-over-water method, are typically implemented using water-tank–based paradigms that exploit the muscle atonia characteristic of REM sleep. In these systems, mice or rats lose postural tone upon entering REM sleep and consequently fall into the water [13], [14], [15], [16]. While these approaches are effective in suppressing REM sleep, they suffer from notable limitations. Most importantly, continuous physical disturbance and water exposure can induce a high level of corticosterone and anxiety, potentially activating stress-related neural and hormonal pathways [14], [17]. Such nonspecific effects may confound experimental outcomes, making it difficult to attribute observed physiological or behavioral changes solely to REM sleep deprivation.

Owing to the limitations of traditional RSD paradigms, a variety of automated approaches based on EEG/EMG monitoring have been proposed in recent years [18], [19], [20]. These methods typically record EEG/EMG signals to identify sleep states in real time and deliver targeted stimulation when REM or NREM sleep is detected, thereby improving sleep-stage specificity and experimental control. Building on these advances, several automated sleep deprivation systems have been developed and commercialized [21], including platforms that employ EEG/EMG-triggered or randomly timed mechanical stimulation. Although these systems provide greater specificity than traditional approaches, many necessitate removal of animals from their home cages and transfer to specialized apparatuses, which may introduce additional environmental stress and perturb natural sleep patterns. Furthermore, commercially available platforms are frequently costly, closed-source, and constrained in their ability to be customized, integrated with external hardware, or extended using user-defined algorithms. Together, these constraints highlight a critical need for open, modular, and cost-effective sleep deprivation systems capable of operating within the home-cage environment and accommodating diverse experimental requirements.

In this work, we propose a home-cage–compatible shaking platform, in which the animal’s original cage is placed directly onto a motorized platform. Upon detection of REM sleep, controlled shaking is applied to selectively disrupt REM episodes, minimizing unnecessary disturbance during other sleep stages and preserving a familiar housing environment. Importantly, the objective of this system is to reduce overall REM sleep duration rather than to eliminate REM entirely. Because detection is state-dependent, brief REM episodes may precede intervention. This does not conflict with the experimental goal, which is to reduce cumulative REM time rather than eliminate REM instantaneously. Because sleep scoring operates at second-level temporal resolution, millisecond-level interruption is unnecessary. The system is designed to achieve sustained reduction of REM sleep over extended periods.

In addition to the advantages described above, the system is built upon the Open Ephys acquisition framework. Open Ephys is an open-source electrophysiological recording platform that is widely used in neuroscience research due to its modular architecture, community-driven development, and demonstrated robustness in long-term and high-channel-count recordings [22], [23], [24]. Building the present RSD system on the Open Ephys platform enables seamless integration with an established open-source ecosystem while ensuring reproducibility, scalability, and accessibility.

In summary, the hardware presented in this work contributes to the field in three key ways. First, it enables closed-loop REM sleep disruption directly within the home-cage environment, minimizing environmental stress and preserving natural housing conditions. Second, it achieves state-specific intervention through real-time EEG-based classification, improving sleep-stage specificity compared with traditional water-based paradigms. Third, by building on the Open Ephys open-source ecosystem, the system provides affordability, modularity, and extensibility, facilitating reproducibility and broad adoption. Together, these features distinguish the proposed platform from conventional and commercial REM sleep deprivation systems and highlight its practical value for scalable closed-loop neuroscience experiments.

## 3 Hardware description

### 3.1 Overall system architecture

**Fig. 1:**
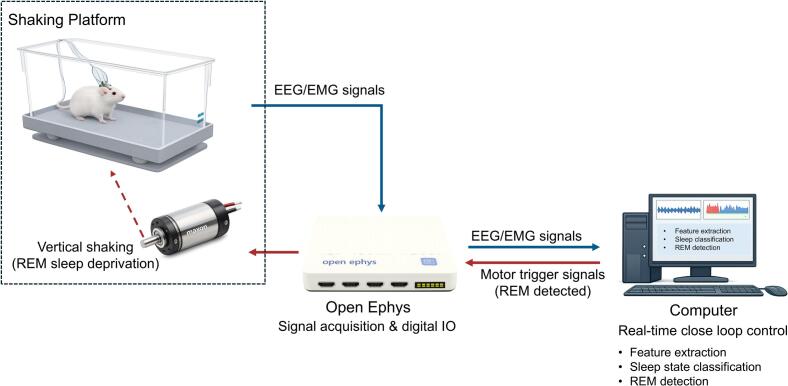
Schematic overview of the closed-loop automated REM sleep deprivation system. EEG and EMG signals from freely behaving mice are acquired via the Open Ephys acquisition board and streamed to a host computer for real-time feature extraction and sleep-state classification. Upon REM detection, the Open Ephys digital I/O interface outputs a TTL signal to the servo motor controller, activating the shaking platform.

The proposed closed-loop automated sleep deprivation system consists of three interconnected subsystems: (i) an EEG/EMG acquisition subsystem, (ii) a real-time sleep-state detection and control subsystem, and (iii) a mechanical stimulation subsystem. An overview of the system architecture is shown in Fig. 1. EEG and EMG signals from freely behaving mice are acquired using the Open Ephys system and streamed to a host computer for low-latency, real-time processing. The control software continuously extracts signal features and performs online sleep-state classification, forming the basis for closed-loop operation. When a target sleep state (e.g., REM sleep) is detected, the system generates digital trigger signals that are sent back through the Open Ephys digital I/O interface to actuate the shaking platform, inducing controlled vertical oscillatory motion for sleep deprivation. This signal flow establishes a fully automated closed-loop framework, enabling state-dependent intervention within the temporal resolution of sleep-state detection.

### 3.2 Signal acquisition and REM detection logic

**Fig. 2:**
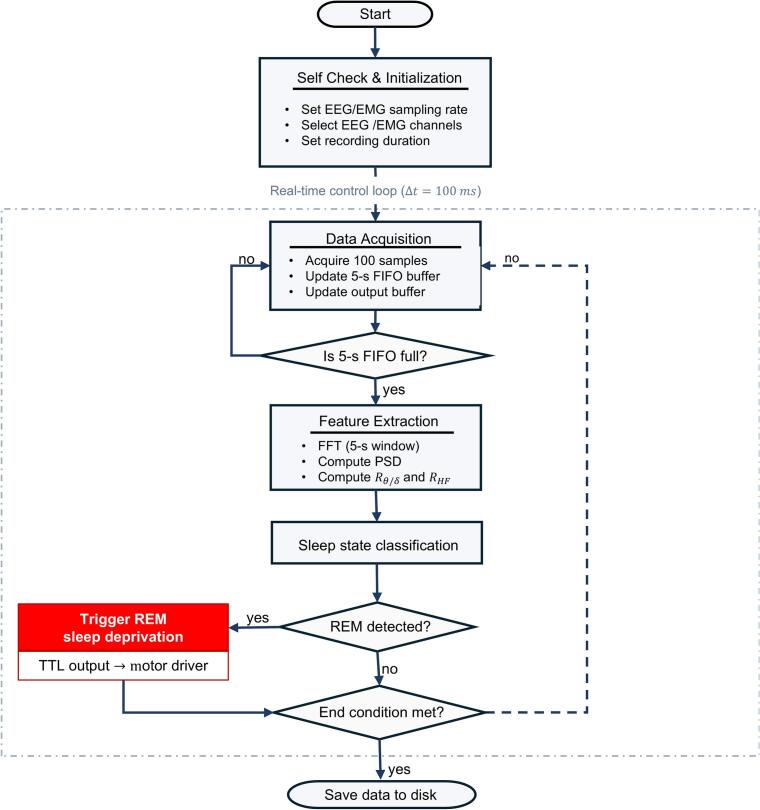
Workflow of the real-time closed-loop REM sleep deprivation system. The system operates in a fixed-time control loop with a 100 ms update interval. EEG signals are continuously acquired at 1 kHz and buffered in a 5 s sliding window for real-time analysis. At each update step, the detection pipeline performs signal preprocessing, spectral feature extraction using FFT, and threshold-based sleep-state classification based on theta/delta ratio and high-frequency power. When REM sleep is detected, a TTL trigger is generated within the same control cycle to initiate mechanical stimulation via the motor driver, forming a closed-loop feedback mechanism.

The overall signal-processing and control workflow is illustrated in Fig. 2. The real-time REM detection pipeline consists of four sequential stages: (i) signal acquisition and preprocessing, (ii) spectral feature extraction, (iii) threshold-based state classification, and (iv) closed-loop actuation.

#### 3.2.1 Signal preprocessing

EEG and EMG signals were continuously acquired using an Open Ephys acquisition board interfaced with an Intan RHD-series headstage (Intan Technologies, USA). Analog-to-digital conversion was performed on the Intan headstage using a 16-bit ADC with a ±5 mV input range. Signals were sampled at 1 kHz and streamed to a host computer for real-time processing. The analog bandpass filter of the recording system was configured to 0.5–500 Hz, consistent with the Nyquist frequency of the selected sampling rate. Although the Intan amplifier supports programmable low-pass filtering up to 7.5 kHz, the effective recording bandwidth in the present configuration was limited to 500 Hz. No additional digital bandpass filtering was applied during online processing.

#### 3.2.2 Spectral feature extraction

EEG spectral features were extracted using real-time power spectral density (PSD) analysis. The PSD was computed from the time-domain EEG signal using a Fast Fourier Transform (FFT)–based approach. A 5 s sliding window (5000 samples at 1 kHz sampling rate) was applied to the continuous EEG signal. Prior to FFT computation, a Hanning window was applied to reduce spectral leakage. The windowed signal was zero-padded to 10,000 points, resulting in a frequency resolution of 0.1 Hz per FFT bin.

For each window, discrete PSD function ${p(f}_{k}$) was estimated and two spectral features were derived from the numerical integration over specified frequency bands, as shown in Eqs.(1) and (2). The first feature is $R_{\theta /\delta }$ (unitless), which is the theta power (4–8 Hz) ratio to the delta power (0.5–4 Hz). $R_{\theta /\delta }$ is used to differentiate REM from NREM sleep. The other feature is $R_{\mathrm{HF}}$ (unitless), which is calculated over 90–200 Hz and normalized by the total EEG energy in the 0.5–500 Hz frequency range. $R_{\mathrm{HF}}$ is used to distinguish wakefulness from sleep states.

(1) $$R_{\theta /\delta }=\sum_{f_{k}\in \left[4,8\right]}p\left(f_{k}\right)\Delta f/\sum_{f_{k}\in \left[0.5,4\right]}p\left(f_{k}\right)\Delta f$$

(2) $$R_{\mathrm{HF}}=\sum_{f_{k}\in \left[90,200\right]}p\left(f_{k}\right)\Delta f/\sum_{f_{k}\in \left[0.5,500\right]}p\left(f_{k}\right)\Delta f$$

#### 3.2.3 Threshold-based REM classification

Once these features are computed, brain-state classification is performed using a threshold-based comparison method. The decision logic for classifying wake, NREM, and REM sleep states is defined by the rule set described in Eq. (3).

(3) $$\mathrm{State}\left(t\right)=\left\{\begin{array}{ccc} \mathrm{REM}, & R_{\theta /\delta }\geq \tau_{\theta /\delta }, & R_{\mathrm{HF}}<\tau_{\mathrm{HF}}\\ Wake, & R_{\mathrm{HF}}\geq \tau_{\mathrm{HF}}\\ NREM, & R_{\theta /\delta }<\tau_{\theta /\delta }, & R_{\mathrm{HF}}<\tau_{\mathrm{HF}} \end{array}\right)$$

In Eq. (3), the thresholds $\tau_{\theta /\delta }$ (unitless) and $\tau_{\mathrm{HF}}$ (unitless) are obtained through a baseline calibration procedure performed for each animal prior to the closed-loop experiment. During this baseline calibration period, EEG and EMG signals are recorded and analyzed to determine subject-specific feature statistics, from which optimal threshold values are derived and subsequently applied for online sleep-state classification.

Each animal has individually calibrated classification thresholds to account for inter-subject variability in EEG and EMG signal amplitudes. Therefore, threshold values are determined separately for each animal using a baseline recording session. First, baseline recording is acquired without intervention. The recorded data are manually scored into three vigilance states: REM sleep, NREM sleep, and wakefulness. For each state, the mean and standard deviation (SD) of the relevant spectral parameters are calculated. The classification thresholds are then defined as:

(4) $$\tau_{\theta /\delta }=mean\left(R_{\theta /\delta }^{\mathrm{REM}}\right)-sd\left(R_{\theta /\delta }^{\mathrm{REM}}\right)$$

(5) $$\tau_{\mathrm{HF}}=mean\left(R_{\mathrm{HF}}^{\mathrm{NREM}}\right)+sd\left(R_{\mathrm{HF}}^{\mathrm{NREM}}\right)$$

where: $R_{\theta /\delta }^{\mathrm{REM}}$ (unitless) represents the theta/delta ratio during manually scored REM epochs and $R_{\mathrm{HF}}^{\mathrm{NREM}}$ (unitless) represents the high-frequency power during manually scored NREM epochs. This adaptive thresholding approach ensures robust classification performance while compensating for inter-animal variability in signal amplitude and electrode impedance. Classification thresholds were determined individually for each animal using statistical features extracted from a 24 h baseline recording acquired without shaker actuation. During closed-loop operation, REM detection was performed prior to motor activation, and epochs during active shaking were not used for threshold estimation or recalibration. Movement-related artifacts increased high-frequency power and were therefore classified as wakefulness rather than REM.

#### 3.2.4 Control cycle, actuation, and data logging

A fixed-length sliding window of 5 s was employed for continuous signal analysis. The window was updated every 100 ms by appending newly acquired EEG samples and discarding the oldest data, corresponding to a step size of 100 ms and an update rate of 10 Hz. This configuration results in a 98% overlap between consecutive windows. At each update step, the refreshed window was processed through the complete detection pipeline, including signal preprocessing, spectral feature extraction, and threshold-based REM classification. The REM recognition procedure required approximately 20 ms per update (Fig. S1, Supplementary Information), allowing each vigilance-state decision to be completed well within the 100 ms update interval. As a result, the vigilance state was re-evaluated every 100 ms, enabling near real-time detection of REM episodes and timely triggering of downstream actuation. When the REM detection criteria were satisfied, a Transistor–Transistor Logic (TTL) trigger signal was generated within the same control-loop iteration and transmitted via the Open Ephys digital I/O interface to the motor controller (servo driver). The latency from REM detection to TTL trigger output was less than 1 ms. Upon receiving a high-level trigger, the motor driver executed a predefined motion profile in velocity-control mode, including a controlled acceleration phase followed by sustained oscillatory platform motion. When the trigger signal returned to low level, motor output ceased. The control loop continued until a predefined termination condition was met. For offline validation and comparison with manually scored sleep stages, classifier outputs were aggregated into 2.5 s epochs, consistent with the epoch duration used by the AccuSleep sleep-scoring software. Because the real-time classifier produced a state estimate every 100 ms, each 2.5 s epoch contained 25 classifier outputs. The final state label for each 2.5 s epoch was determined using a majority-voting rule, whereby the state occurring most frequently among the 25 outputs was assigned as the epoch label. Accordingly, while real-time detection operated at 100 ms temporal resolution, the stored classification results used for offline analysis and comparison with ground-truth labels were organized into 2.5 s epochs.

#### 3.2.5 System workflow overview

Beyond the core detection and control pipeline, the platform implements automated self-check and validation procedures to enhance system robustness and operational safety. These procedures verify hardware connections, confirm stable data acquisition, and test the integrity of the digital triggering pathway before the experiment begins. During system initialization, a short motor actuation test (∼5 s) is also executed to confirm proper operation of the actuation subsystem, with visible platform motion serving as verification prior to enabling closed-loop control. Combined with the real-time signal-processing and closed-loop control modules, the system operates as a unified framework designed for reliable long-term experimentation. A comprehensive overview of the operational logic is presented in the system workflow diagram in Fig. 2.

*For offline validation, real-time classification outputs (100 ms resolution) are aggregated into 2.5 s epochs using a majority-voting rule, ensuring direct comparability with manually scored sleep stages. The loop continues until a predefined termination condition is met, after which all data are saved for analysis.*

### 3.3 Mechanical design and motion characteristics

#### 3.3.1 Mechanical architecture design

**Fig. 3:**
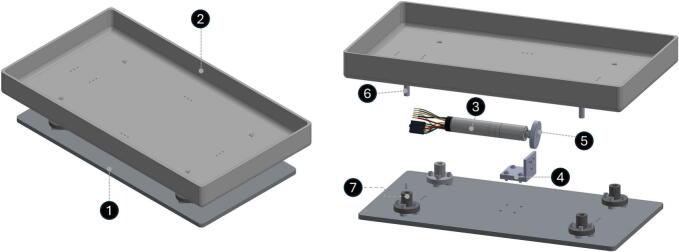
Overall assembly and exploded view of the shaking platform. (1) Base plate, (2) cage carrier, (3) DC motor, (4) motor mounting adapter, (5) elliptical cam, (6) ground shaft, and (7) flanged bushing. The eccentric cam converts motor rotation into controlled vertical oscillatory motion of the cage carrier.

The mechanical subsystem consists of a motor-driven oscillatory platform designed to induce controlled vertical displacement of the animal’s home cage. The cage is placed directly onto a rigid carrier plate mounted on dual linear guide shafts to constrain motion to a single vertical axis while minimizing lateral displacement and unwanted mechanical slack. The overall assembly and major components are illustrated in Fig. 3. The platform consists of a base plate (1), cage carrier (2), DC motor (3), motor mounting adapter (4), elliptical cam (5), ground shaft (6), and flanged bushings (7), as labeled in Fig. 3.

#### 3.3.2 Motor and actuation mechanism

A brushless DC motor drives a custom-made elliptical cam plate to produce vertical motion of the platform. During rotation, the non-circular elliptical profile converts rotational motion into periodic linear displacement, with the displacement amplitude determined by the geometric difference between the ellipse’s major and minor axes. The motor is operated in velocity-control mode using a dedicated servo driver, where the motor rotational speed serves as the primary control input. The specified speed determines the rotational speed of the cam plate through a gear reduction stage and thereby defines the oscillatory behavior of the platform. The acceleration profile governs the dynamic response during startup. Accordingly, the peak-to-peak vertical displacement of the platform can be approximated as

(6) $$d_{\mathrm{pp}}\approx L_{\mathrm{major}}-L_{\mathrm{minor}}$$

where $L_{\mathrm{major}}$ (mm) and $L_{\mathrm{minor}}$ (mm) in Eq. (6) denote the major and minor axis lengths of the elliptical cam, respectively. To ensure reproducibility and facilitate system configuration, the key control settings, hardware specifications, transmission parameters, and kinematic characteristics of the actuation subsystem are summarized in Table 1.

**Table 1:** Summary of motor specifications, transmission configuration, mechanical geometry, and control parameters of the oscillatory actuation system.

Category	Parameter	Value	Unit
Motor specifications	Motor type	Brushless DC	−
	Rated Voltage	24	V
	Nominal speed	43,200	rpm
	Maximum continuous torque	9.1	mNm
Transmission	Gear reduction ratio	103	
Mechanical geometry	Cam profile	Elliptical	
	Major axis length	15	mm
	Minor axis length	10	mm
Control settings	Control mode	speed	
	Target motor speed	8000	rpm
	Target Motor Acceleration	523.6	$\mathrm{rad}/s^{2}$
	Acceleration time	1.6	s
Kinematic characteristics	Peak-to-peak displacement	10	mm
	Cam rotational speed	77.7	rpm
	Oscillation frequency	1.3	Hz

### 3.4 Structural stability, environmental compatibility, and system expandability

Structural stability is achieved using dual linear guide shafts and flanged bushings, which constrain motion to the vertical axis while minimizing friction and lateral instability. A rigid base plate further reduces structural deformation during oscillation and provides a stable mounting platform for the motor assembly.

To minimize thermal and environmental interference, the motor and actuation components are mounted on a base plate that is physically separated from the cage carrier. The cage rests above the mechanical assembly and is mechanically coupled only through guiding structures, thereby limiting heat transfer to the animal environment. In addition, actuation is event-driven and triggered only upon REM detection rather than operating continuously. This intermittent operation reduces cumulative thermal output and minimizes environmental disturbance. Predictive modelling approaches, such as finite element analysis (FEA) and AI-based vibration modelling, have been widely used to evaluate mechanical stability and vibration characteristics in hardware validation studies [25]. In the present work, the mechanical architecture was refined through iterative prototyping and empirical testing rather than predictive simulation. Nevertheless, the modular and well-constrained structural design remains amenable to future integration of predictive modelling tools for vibration analysis and structural optimization.

The system adopts a modular architecture composed of independent functional modules for data acquisition, sleep-stage classification, and motor actuation control. Because these modules are functionally decoupled, replacing the recording hardware requires modification only at the acquisition interface layer, while the REM detection algorithm and motor control logic remain unchanged. Consequently, the system is not restricted to a specific acquisition platform and can be readily adapted to other systems that provide real-time electrophysiological data streaming and standard TTL outputs.

### 3.5 Software implementation, computational performance, and reproducibility

The control software was implemented in C++ and executed on a standard laptop computer (Intel Core Ultra 9 285 V processor, 32 GB RAM, Windows 11, 64-bit). Feature extraction and state classification were benchmarked using high-resolution timing functions. The average computation time per 100 ms update cycle was approximately 20 ms (Fig. S1, Supplementary Information), providing substantial computational margin and ensuring stable real-time performance during long-duration experiments. To ensure transparency and reproducibility, the complete software for the real-time REM sleep classification and closed-loop control system, as well as the offline analysis pipeline used to generate all reported results, is publicly available on the Open Science Framework (OSF) at https://osf.io/r3u5g. Specifically, the OSF repository provides: (i) scripts for real-time EEG-based brain-state classification and closed-loop stimulation, (ii) scripts for per-animal feature and threshold estimation, and (iii) mechanical design files. Together, these materials enable full software-level reproducibility of the real-time pipeline described in this work.

### 3.6 Potential applications and broader utility

• Enables automated REM sleep deprivation experiments in standard home-cage environments while minimizing experimenter intervention and handling-related stress.
• Supports long-term closed-loop sleep manipulation studies investigating memory consolidation, emotional regulation, neurodegeneration, psychiatric disorders, and the effects of pharmacological agents such as opioids on sleep architecture and brain-state regulation.
• Provides a flexible open-source platform that can be adapted for other brain-state–dependent interventions, including sensory stimulation, optogenetic stimulation, and neural activity tagging experiments.
• Offers a reproducible, affordable, and customizable alternative to commercial sleep manipulation systems for neuroscience research and educational laboratories.

## 4 Design files summary

All design and software files required to reproduce the closed-loop REM sleep deprivation system are summarized in Table 2. All files are publicly available via the Open Science Framework and are distributed under the Creative Commons Attribution 4.0 International License to facilitate reuse and reproducibility.

**Table 2:** Design files summary.

Design file name	File type	Open source license	Location of the file
FAB-01	. STEP	CC BY 4.0	Open Science Framework (10.17605/OSF.IO/R3U5G)
FAB-02	. STEP	CC BY 4.0	Open Science Framework (10.17605/OSF.IO/R3U5G)
FAB-03	. STEP	CC BY 4.0	Open Science Framework (10.17605/OSF.IO/R3U5G)
FAB-04	. STEP	CC BY 4.0	Open Science Framework (10.17605/OSF.IO/R3U5G)
FAB-05	. STEP	CC BY 4.0	Open Science Framework (10.17605/OSF.IO/R3U5G)
REM Sleep Detection	.zip	CC BY 4.0	Open Science Framework (10.17605/OSF.IO/R3U5G)
Feature_Extraction	.zip	CC BY 4.0	Open Science Framework (10.17605/OSF.IO/R3U5G)

Specifically, FAB-01 is the base plate used to support the overall mechanical structure, FAB-02 is the mouse cage carrier designed for home-cage-compatible experiments, FAB-03 is the elliptical cam plate that generates periodic vertical motion, FAB-04 consists of precision ground shafts used for motion guidance and stabilization, and FAB-05 is the motor mounting adapter used to connect and secure the servo motor assembly. These files are provided as STEP-format 3D CAD models for mechanical fabrication and system assembly. The “REM Sleep Detection” archive contains the source code for real-time EEG-based REM sleep detection and closed-loop motor control. The “Feature_Extraction” archive provides scripts for EEG spectral feature extraction, classification-threshold estimation, and offline sleep-state analysis.

## 5 Bill of materials summary

All hardware components required to assemble and operate the closed-loop REM sleep deprivation system are summarized in Table 3. Custom-fabricated mechanical parts are clearly distinguished from commercially available components using consistent designators (FAB: fabricated parts; MECH: standard mechanical components; ELEC: electrical components). For each component, the table summarizes the manufacturer, part number or specification, quantity, and cost, where applicable, to support system reproducibility. General-purpose laboratory equipment, such as a standard personal computer used to run the Open Ephys graphical user interface and perform real-time signal processing, is not included in the bill of materials.

**Table 3:** Bill of materials for the closed-loop REM sleep deprivation system.

Designator	Component	Manufacturer	Part number/Specification	Qty	Cost
FAB-01	Base plate	Custom (CNC machined)	See CAD files; tolerance ±0.05 mm	1	$26.16
FAB-02	Mouse cage carrier	Custom (CNC machined)	See CAD files; tolerance ±0.05 mm	1	$211.07
FAB-03	Elliptical cam plate	Custom (CNC machined)	See CAD files; tolerance ±0.05 mm	1	$22.75
FAB-04	Precision ground shaft	Custom (CNC machined)	See CAD files; tolerance ±0.05 mm	4	$51.8
FAB-05	Motor mounting adapter	Custom (CNC machined)	See CAD files; tolerance ±0.05 mm	1	$34.51
MECH-01	Linear ball bearing with round flange	Generic	See CAD files; tolerance ±0.05 mm	4	$12.00
MECH-02	M1.2 × 0.2 × 5 mm screw	Generic	ISO metric screw, M1.2 × 0.2 × 5 mm, stainless steel	12	$1.20
MECH-03	M2 × 0.4 × 5 mm screw	Generic	ISO metric screw, M2 × 0.4 × 5 mm, stainless steel	4	$0.40
MECH-04	M3 × 0.5 × 5 mm screw	Generic	ISO metric screw, M3 × 0.5 × 5 mm, stainless steel	4	$2.00
MECH-05	M3 × 0.5 × 12 mm screw	Generic	ISO metric screw, M3 × 0.5 × 12 mm, stainless steel	16	$8.00
MECH-06	M4 hex nut	Generic	ISO metric screw, M4 hex nut, stainless steel	16	$2.00
ELEC-01	HDMI cable	Generic	HDMI cable, 1 m length	1	$8.00
ELEC-02	DC motor with gearbox and encoder	Maxon	Part number: B82C73D8A27A; ECXSP16L motor with GPX16HP gearbox; see datasheet in repository	1	$584.25
ELEC-03	Maxon Servo controller	Maxon	Part number: 409,510	1	$337.25
ELEC-04	Open Ephys acquisition board	Open Ephys	Part number: OEPS-9029	1	$3780.00
ELEC-05	Open Ephys I/O board	Open Ephys	Part number: OEPS-6501	1	$99.00
ELEC-06	EEG/EMG headstage (Open Ephys compatible)	Intan	Part number: C3334	1	$805.00
ELEC-07	SPI tether cable for headstage	Intan	Part number: C3203	1	$215.00

## 6 Build instructions

The construction of the closed-loop REM sleep deprivation system is divided into two main parts: (i) mechanical assembly of the shaking platform, and (ii) electrical wiring and signal connections between the control electronics and the actuator. All components referenced below correspond to the design file summary and bill of materials (BOM) summary sections, where dimensioned CAD drawings are also available.

### 6.1 Mechanical assembly of the shaking platform

**Fig. 4:**
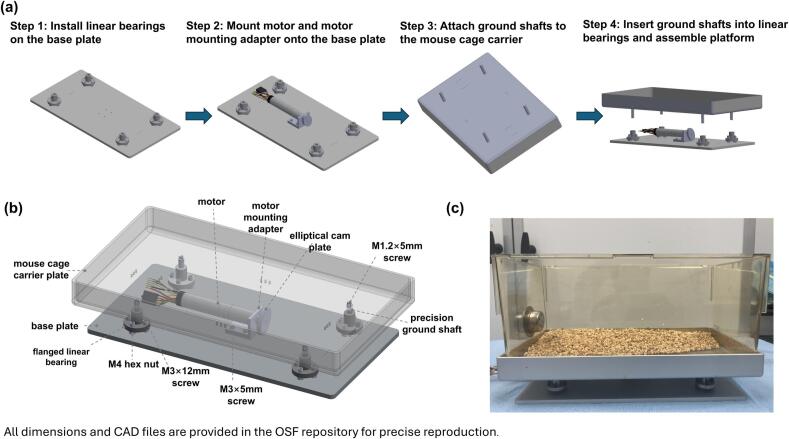
Assembly of the shaking platform.(a) Step-by-step assembly workflow, including installation of linear bearings, motor mounting, attachment of ground shafts, and final platform assembly. (b) Annotated CAD view highlighting key components and fasteners. (c) Photograph of the assembled device. All dimensions and detailed CAD drawings are provided in the OSF repository to enable precise and reproducible assembly.

An overview of the assembled system is shown in Fig. 4. The assembly process for the shaking platform module is summarized below as step-by-step instructions:

Step 1: Install the flanged linear bearing onto the base plate using M3 × 12 mm socket head screws. Both components are aligned with the pre-tapped M3 holes in the base plate. An M4 hex nut is inserted between the bearing flange and the base plate to serve as a spacer and increase vertical clearance.

Step 2: Mount the motor onto the motor mounting plate using M2 × 5 mm socket head screws and securely attach the custom elliptical cam plate to the motor shaft. And finally install motor mounting adapter onto the base plate using M3 × 5 mm socket head screws.

Step 3: Attach the four precision ground shafts to the mouse cage carrier plate using M1.2 × 5 mm screws, ensuring perpendicular alignment to the mouse cage carrier plate.

Step 4: Insert the ground shafts of the cage carrier plate into the flanged linear bearings. The carrier plate rests on the elliptical cam under gravity, enabling vertical oscillatory motion during motor actuation.

### 6.2 Electrical wiring and signal connections

**Fig. 5:**
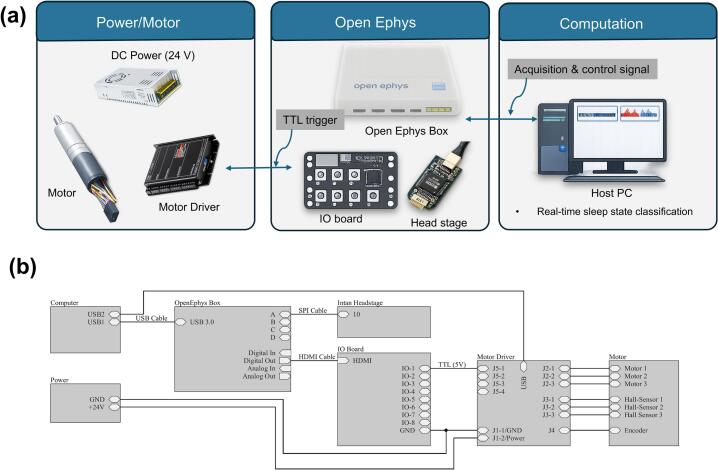
System-level architecture of the closed-loop REM sleep deprivation system integrating EEG/EMG acquisition, real-time sleep state classification, and motor actuation. (b) Component-level electrical wiring schematic illustrating the direct 5 V TTL connection between the Open Ephys IO board and the motor driver, including power and ground routing.

The actuation module is based on a commercially configured Maxon drive, consisting of an ECXSP16L BL KL A STD 24 V brushless motor, a GPX16HP 103:1 gearhead, and an ENX16 EASY INT COMM 1024IMP encoder. The motor is rated as 24 V operation with a nominal continuous current of 2.05 A and a power rating of 40 W. The motor is controlled using a commercial Maxon ESCON 50/5 servo controller powered by a 24 V DC supply. The controller supports external 5 V TTL triggering via its digital input interface. No custom-designed motor driver or PCB circuitry was developed in this study. The electrical wiring of the closed-loop REM sleep deprivation system establishes the connections between the control electronics, power supply, and actuator. The system-level architecture and the detailed electrical wiring schematic are shown in Fig. 5a and b, respectively. The wiring process follows the functional signal flow of the system and is described below.

• The host PC is connected to the Open Ephys acquisition box via a USB 3.0 cable, enabling bidirectional communication for real-time data acquisition and control signal transmission.
• The Intan headstage is connected to the Open Ephys system using the dedicated data cable. EEG and EMG signals recorded from the animal are transmitted through this interface and assigned to the corresponding input channels of the Open Ephys acquisition box.
• The Open Ephys acquisition box is connected to the IO board via an HDMI cable. TTL trigger signals generated by the Open Ephys system under predefined sleep-state conditions are routed to the IO board. As illustrated in Fig. 5b, the digital output channel (IO-1) of the IO board provides a direct 5 V TTL-level signal, which is connected to the digital input (IO-1) of the motor driver using a BNC-terminated coaxial cable. The Open Ephys system and the motor driver share a common ground reference through this interface. No additional signal conditioning or custom circuitry is used in this connection.
• The motor driver is powered by a 24 V DC power supply connected to the motor driver power connector (V+ and GND). Correct polarity is verified before system operation. The motor output terminals of the motor driver (J2 connector) are connected to the motor leads, delivering the motor driving signals required for actuator operation. The encoder connector (J4) of the motor driver is connected to the motor encoder interface to enable position and motion feedback. The Hall sensor connector (J5) of the motor driver is connected to the corresponding Hall sensor outputs of the motor, providing additional feedback signals for motor control. The detailed connector pin mapping is summarized in Table S1 (Supplementary Information).

## 7 Operation instructions

The REM sleep deprivation system is operated according to a standardized workflow to ensure reproducibility across animals and experimental sessions.

### 7.1 Preparation and habituation

All experiments were conducted using Ai14-Trap2 transgenic mice on a C57BL/6J background (approximately 4.5 months old). For tethered EEG/EMG recordings, mice were surgically implanted with two EEG and two EMG electrodes. EEG electrodes were constructed by wrapping 0.002-inch stainless steel wire around skull screws positioned over the left and right cortex (AP −3.5 mm, ML 3 mm; AP + 1.5 mm, ML 1.5 mm). EMG electrodes were formed by inserting 0.003-inch stainless steel wire into the neck musculature. A single skull screw positioned over the cerebellum (AP −6.0 to −6.7 mm, ML 0 mm) was used as both the reference and ground electrode for all recordings. Leads were soldered to a pin header and secured with dental cement. Advanced low-noise electrode technologies have been proposed to further improve signal reliability and recording comfort in bioelectrical systems [26]. Although the present implementation employs stainless steel screw-based electrodes commonly used in rodent sleep research, the modular acquisition architecture is compatible with alternative low-noise electrode solutions. In addition, the per-animal calibration procedure mitigates variability in signal amplitude and noise characteristics, ensuring robust sleep-state detection across recordings.

Following surgery, mice were allowed to recover for at least two weeks prior to experimentation. Before baseline recording and threshold calibration, mice were placed on the shaking platform within the sleep recording chamber and connected to the EEG/EMG recording system. A habituation period of at least 24 h was provided to minimize stress-related artifacts and allow adaptation to the recording environment.

### 7.2 Ground-truth labeling, threshold estimation, and validation

To generate ground-truth labels for threshold estimation and performance evaluation, baseline EEG recordings were acquired at a sampling rate of 1000 Hz without activation of the shaking platform. In the present study, each animal underwent a 24 h baseline recording to capture representative wake, NREM, and REM sleep states. The recordings were then scored offline using validated sleep-state labeling software (AccuSleep) [27], which segmented the data into 2.5 s epochs and assigned each epoch to Wake, NREM, or REM. These offline labels served as the ground truth for subsequent threshold estimation and validation.

Because EEG characteristics vary across individual animals, detection thresholds were determined on a per-animal basis. Using the ground-truth labels obtained from offline scoring, the thresholds for $\tau_{\theta /\delta }$ and $\tau_{\mathrm{HF}}$ were estimated according to the method described above. The resulting threshold values were then loaded into the real-time closed-loop control software.

A separate validation session was subsequently performed using online sleep-state classification. The classification results produced by the real-time system were compared with the offline ground-truth labels to assess detection performance. If the classification accuracy met predefined acceptance criteria, the system proceeded to closed-loop REM intervention. Otherwise, the detection parameters were refined, and when necessary, additional baseline data were acquired to re-estimate thresholds and improve state separation reliability.

### 7.3 Closed-loop experimentation

Closed-loop REM manipulation is enabled only after successful validation. For REM sleep manipulation experiments, eight mice were randomly assigned to two groups: a REM sleep deprivation group and a REM sleep recovery group. Mice in the REM sleep deprivation group received 4-hydroxytamoxifen (4-OHT) administration prior to the deprivation experiment, whereas mice in the REM sleep recovery group received 4-OHT administration after completion of the deprivation period. Closed-loop REM sleep deprivation was then performed for 48 h, during which platform motion was automatically triggered upon detection of REM sleep. Following completion of the deprivation period, mice were returned to standard housing conditions. Ten days after the end of REM sleep deprivation, mice were deeply anesthetized and a transcardial perfusion was performed. Brains were collected, fixed, and processed for histological analysis. All animal procedures were approved by the Institutional Animal Care and Use Committee and were conducted in accordance with relevant guidelines.

## 8 Validation and characterization

### 8.1 Validation of real-time sleep state classification performance

**Fig. 6:**
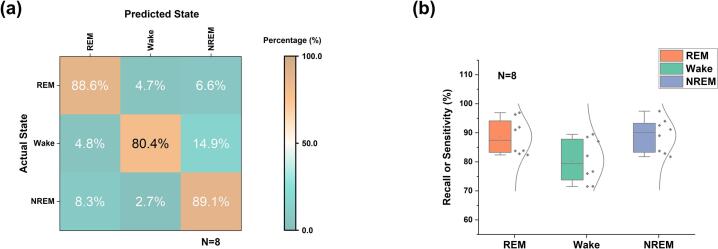
Performance of real-time sleep state classification using EEG-only features. (a) Confusion matrix summarizing classification performance across all animals (N = 8). Rows represent true labels (actual state), and columns represent predicted labels (REM, Wake, NREM). Values indicate the percentage of epochs within each true class. High values along the diagonal indicate correct classifications. (b) Distribution of per-animal recall (sensitivity) for each sleep state (REM, Wake, NREM). Boxplots show median and interquartile range, with individual data points overlaid (N = 8).

To quantitatively evaluate the performance of the proposed real-time sleep state classifier, we analyzed its ability to discriminate REM, Wake, and NREM states across multiple animals. As shown in Fig. 6a, the confusion matrix shows strong diagonal dominance, indicating high state-specific classification accuracy. Specifically, 88.6% of REM epochs, 80.4% of Wake epochs, and 89.1% of NREM epochs were correctly classified, demonstrating reliable separation between the three vigilance states. Misclassifications were primarily observed between Wake and NREM, with 14.9% of NREM epochs incorrectly labeled as Wake and 2.7% of Wake epochs misclassified as NREM, reflecting the known spectral overlap between these two states. In contrast, REM sleep was rarely confused with Wake or NREM, indicating robust detection of REM-specific EEG features. Per-animal confusion matrices (Fig. S2) further demonstrate the consistency of classification performance across individual subjects.

The per-animal recall distributions shown in Fig. 6b further demonstrate the robustness of the classifier. Across animals, REM and NREM sensitivities were consistently high, while Wake showed slightly larger variability but remained within an acceptable range for closed-loop applications. Consistent with these observations, the quantitative performance metrics listed in Table S2 (Supplementary Information) show high recall and F1-scores across subjects. These results confirm that the proposed system provides stable and reproducible real-time sleep state detection, which is essential for reliable automated REM sleep deprivation.

### 8.2 Experimental validation of REM sleep deprivation

**Fig. 7:**
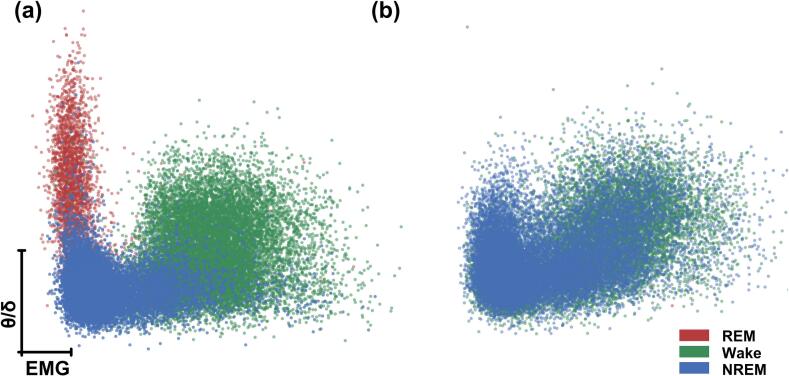
State-space representation of sleep stages under control and REM sleep deprivation conditions. Two-dimensional distributions of EEG (θ/δ) ratio versus EMG activity for a representative mouse during **(a)** baseline control (24 h) and **(b)** closed-loop REM sleep deprivation (24 h). Each point represents a single analysis epoch and is color-coded by vigilance state (REM, Wake, and NREM). Under baseline conditions, REM epochs form a distinct cluster characterized by high θ/δ ratio and low EMG activity. During REM sleep deprivation, this REM cluster is markedly reduced, with points shifting toward Wake and NREM regions, indicating effective closed-loop suppression of REM sleep while preserving the overall structure of non-REM and wake states.

Under control conditions, REM, NREM, and Wake states occupy well-separated regions in the feature space defined by EMG amplitude and the EEG theta-to-delta power ratio (θ/δ), indicating that the selected features provide stable and physiologically meaningful discrimination of sleep states (Fig. 7a). REM sleep is consistently associated with low EMG activity and elevated θ/δ values, forming a distinct and compact cluster that enables reliable real-time detection. Following 24 h of closed-loop REM sleep deprivation, REM-associated data points are markedly reduced, accompanied by a systematic reorganization of the feature space (Fig. 7b). The disappearance of the REM cluster and the redistribution of NREM and Wake states toward regions typically occupied by REM sleep demonstrate that the observed changes are not due to misclassification or random variability but reflect genuine alterations in sleep-state dynamics induced by the intervention. These feature-space changes are consistent with the raw EEG/EMG signals shown in Fig. S3 (Supplementary Information), where REM episodes are substantially suppressed during deprivation while NREM and Wake states dominate. These results validate the effectiveness and robustness of the proposed hardware–software framework for continuous, real-time sleep-state monitoring and closed-loop manipulation. The ability of the system to capture both stable baseline state separation and dynamic state-space reconfiguration following intervention highlights its suitability for reproducible and long-duration sleep experiments.

**Fig. 8:**
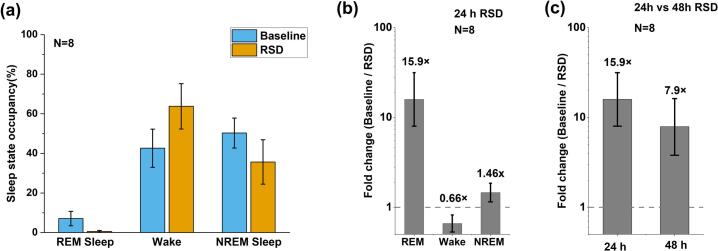
Quantification of REM sleep deprivation across animals. (a) Sleep state occupancy under baseline conditions and during 24-h REM sleep deprivation (RSD). Bars represent the percentage of time spent in REM, Wake, and NREM states, showing a pronounced reduction of REM sleep and a corresponding increase in wakefulness during RSD. (b) Baseline-to-RSD occupancy ratio for each vigilance state during the 24-h deprivation protocol. Ratios were computed per animal and summarized as geometric means; error bars indicate 95% confidence intervals. REM exhibits a large ratio, demonstrating selective suppression, whereas Wake and NREM remain near unity. (c) Baseline-to-RSD ratio for REM sleep during 24-h and 48-h deprivation. Although suppression is partially reduced at 48 h, REM sleep remains strongly suppressed relative to baseline, indicating robust long-term closed-loop control. Statistical significance was assessed using paired Wilcoxon signed-rank tests (n = 8).

To quantify the efficacy and selectivity of closed-loop REM sleep deprivation, we first analyzed sleep-state occupancy under the 24-h RSD condition, as shown in Fig. 8a. The corresponding sleep state distributions for individual mice are provided in Table S3 (Supplementary Information), demonstrating consistent effects across the cohort (n = 8 mice). Under baseline conditions, REM sleep accounted 7.09% ± 3.63% (mean ± SD) of total recording time, whereas during RSD it was reduced to 0.55% ± 0.50%, accompanied by a compensatory increase in wakefulness and a modest decrease in NREM sleep.

To more directly assess the selectivity of the intervention, we computed the baseline-to-RSD fold-change on a per-animal basis for each vigilance state on a per-animal basis and summarized these values using geometric means. According to Fig. 8b, REM sleep exhibited a 15.9-fold reduction (95% CI: 8.0–31.5), corresponding to a >90% suppression relative to baseline. In contrast, wake and NREM showed much smaller changes (0.66-fold and 1.46-fold, respectively), indicating that the closed-loop system selectively suppresses REM sleep rather than broadly disrupting overall sleep architecture. These fold-change metrics define the extent of off-target disturbance, demonstrating that the intervention selectively suppresses REM sleep while largely preserving non-REM architecture. Statistical comparisons were performed on per-animal values using paired Wilcoxon signed-rank tests (n = 8), confirming that the reduction in REM sleep was highly significant (p < 0.001).

We further examined the stability of REM suppression over longer durations by comparing 24-h and 48-h RSD protocols, as shown in Fig. 8c. At 48 h, REM sleep remained strongly suppressed (7.9-fold reduction, 95% CI: 3.8–16.2), although the magnitude was reduced compared with the 24-h condition. This partial attenuation likely reflects increased homeostatic REM pressure and elevated arousal thresholds during prolonged deprivation. Consistent with this interpretation, we occasionally observed that actuator motion persisted for several seconds after REM detection without immediately terminating ongoing REM bouts during later stages of deprivation. This observation indicates that the behavioral effect of mechanical perturbation is probabilistic and state-dependent rather than deterministic at the level of individual triggers.

Importantly, the primary outcome of interest in this study is the aggregate reduction in total REM duration across the closed-loop session, rather than the immediate termination probability of individual REM bouts. Despite reduced per-event efficacy under high sleep pressure, the sustained and statistically significant reduction in total REM sleep at both 24 h and 48 h demonstrates robust closed-loop control over extended time scales.

These findings were consistent with qualitative observations obtained in additional mouse genotypes tested in our laboratory, suggesting that the closed-loop framework is not strain-specific. Because detection thresholds are determined on a per-animal basis, the system can accommodate inter-strain variability in EEG characteristics while maintaining selective REM suppression.

### 8.3 REM sleep-active melanin-concentrating hormone (MCH) neurons are activated during recovery sleep rebound after RSD

**Fig. 9:**
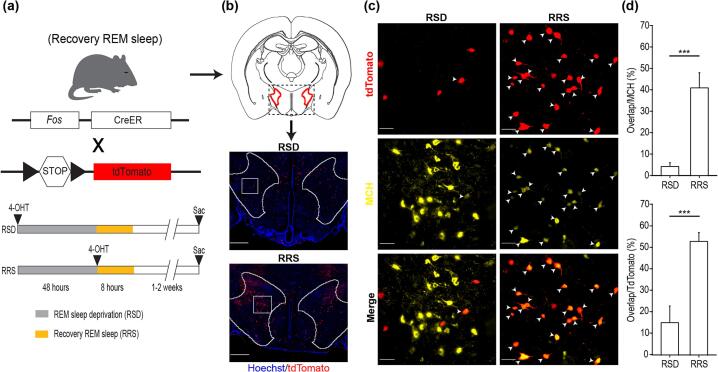
MCH neurons are active during recovery sleep rebound after RSD. (a) Experimental schematic. Mice expressing CreER under the Fos promoter are crossed with Cre-inducible reporter mice expressing tdTomato. Each mouse undergoes 48 h of RSD and undergo 8 h of REM recovery sleep (RRS); 4-OHT was injected immediately before RSD or RRS to label NREM/wake- or REM-active neurons, respectively. Mice were sacrificed 1–2 weeks after. (b) tdTomato expression in the LHA (red outline). Top, coronal diagram of the mouse brain. Bottom, images of tdTomato + neurons in RSD and RRS mice at anterior posterior (AP) −1.70 mm. Blue, Hoechst. Scale bar, 500 μm. (c) Immunohistochemistry of MCH in the LHA. Images taken from the area within the white square in (b). Arrowheads indicate cells co-labeled with tdTomato and MCH. Scale bar, 50 μm. (d) Bar graphs showing the difference in the percentage of overlap between MCH positive and tdTomato positive neurons with the total amount of either MCH neurons (top) or tdTomato positive neurons (bottom) in the LHA. n = 5 each group; error bar, ±SEM; two-tailed Welch’s *t*-test, ***P < 0.005. (For interpretation of the references to colour in this figure legend, the reader is referred to the web version of this article.)

Examination of the immediate early gene (IEG), c-fos, is the most widely used method for studying neuronal activity and immunohistochemically labelling of the active cells in neuroscience [[28], [29]]. In particular, recovery sleep rebound after RSD induces robust c-fos expression in REM-active neurons [[14], [30], [31], [32]]. Based on this strategy, we dissected out the REM-active neurons by using a recently developed activity-dependent genetic labeling technique [[33], [34]], which allows us to permanently label Fos-expressing REM-active neurons with great temporal resolution. We crossed an *iFos-TRAP* transgenic mouse line, *TRAP2* that expresses tamoxifen-inducible Cre recombinase under the Fos promoter (JAX # 030323) [35] with *Ai14* reporter line expressing tdTomato (JAX # 007914) [36]. Mice were divided into two groups (n = 5 each group). In the first group, we paired an i.p. injection of 4-hydroxytamoxifen (4-OHT) with 48 h of RSD. This allows the Cre estrogen receptor (CreER) expressed in NREM- or wake-active neurons to enter the nucleus, leading to permanent expression of tdTomato (Fig. 9a). In the second group, we paired a 4-OHT injection with 8 h of recovery REM sleep (RRS; following 48 h of RSD) to label REM-active neurons with tdTomato. Brains were sectioned into 50 mm coronal slices using a cryostat (RWD Life Science). Immunofluorescence was performed using the following primary antibodies: rabbit anti-melanin-concentrating hormone (MCH) antibody (*Phoenix* Pharmaceuticals, #H-070–47; 1:250) and mouse anti-red fluorescence protein (RFP) antibody (Rockland Immunochemicals, # 200–301-379, 1:250), and the following secondary antibodies: Alexa Fluor 647 goat anti-rabbit IgG (Thermo Fisher, #A-21245; 1:500) and Alexa Fluor 594 goat anti-mouse IgG (Thermo Fisher, #A-11005; 1:500), and Hoechst (Thermo Fisher, #33342; 1:10,000). Fluorescence images were taken using a VS200 slide scanner (Evident Scientific). QuPath was used to analyze the cells within the lateral hypothalamus area (LHA). We observed tdTomato expression in the LHA of RRS mice (Fig. 9b). Immunohistochemical staining showed that most of the tdTomato-labeled LHA neurons in the RRS mice were MCH neurons (Fig. 9c and d), which have been shown to be maximally active during REM sleep [37], respond to REM sleep deprivation, and integrate homeostatic sleep drive [[14], [38], [39], [40]].

## Ethics statements

All animal procedures have been approved by the University of Nebraska Medical Center’s Institutional Animal Care and Use Committee (UNMC-IACUC) and are in accordance with the eighth edition of the National Research Council's Guide for the Care and Use of Laboratory Animals, the requirements of the Public Health Service Grants Administration Manual, and UNMC-IACUC Animal Welfare Assurance, Guidelines and Policies.

## CRediT authorship contribution statement

**Ruilin Yang:** Writing – original draft, Visualization, Validation, Software, Formal analysis, Data curation, Conceptualization. **Wyatt D. Morse:** Writing – review & editing, Validation, Data curation. **Peng Zhong:** Writing – review & editing, Supervision.

## Declaration of competing interest

The authors declare that they have no known competing financial interests or personal relationships that could have appeared to influence the work reported in this paper.

## Appendix A Supplementary data

Supplementary Data 1 Supplementary material includes additional experimental data, supplementary figures and tables, and supplementary analyses related to real-time REM detection, and REM sleep deprivation experiments.
