# Supplementary material for: Development of an automated REM sleep deprivation device for mice in neuroscience research
Source: HardwareX. 2026 May 23;26:e00797. doi: 10.1016/j.ohx.2026.e00797 (PMC13226955; doi:10.1016/j.ohx.2026.e00797)
Supplement: Supplementary Data 1 — Supplementary material includes additional experimental data, supplementary figures and tables, and supplementary analyses related to real-time REM detection, and REM sleep deprivation experiments. [file mmc1.docx]

**Supplementary Materials**

**S1. Latency of real-time REM detection**

To evaluate the temporal performance of the real-time REM detection algorithm, we quantified the detection latency across all trials. Latency was defined as the time elapsed between the availability of the signal window and the generation of the detection decision within the closed-loop system. As shown in Fig. S1, the latency distribution is tightly clustered, with a median of approximately 20 ms and a relatively narrow interquartile range. A small number of outliers with higher latency were observed, likely due to occasional processing or communication delays. Importantly, the majority of detections fall within a consistent time window, indicating stable and reliable real-time operation. These results confirm that the system achieves low-latency performance compatible with the 100 ms update cycle used in the online detection framework, and that the computational overhead does not introduce significant temporal variability across trials.


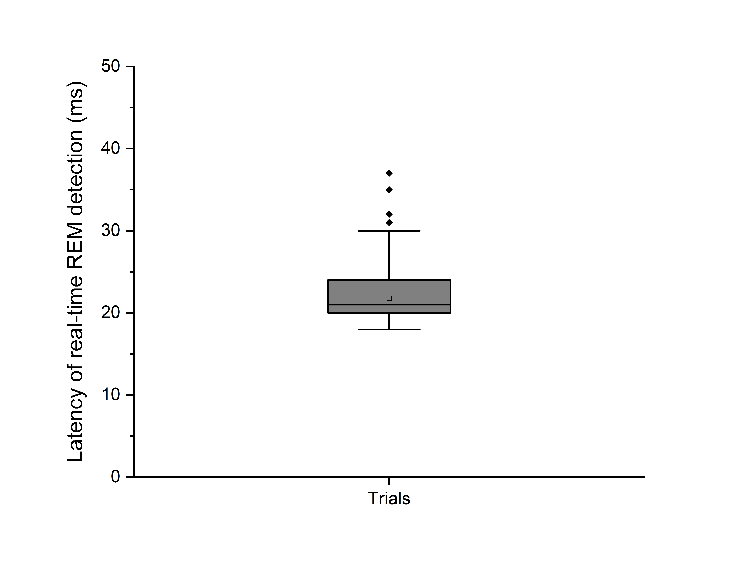


Fig. S1. Distribution of real-time REM detection latency across trials. Box-and-whisker plot summarizing the latency of the real-time REM detection algorithm across all trials. The central line indicates the median, the box represents the interquartile range (IQR), and whiskers extend to 1.5× IQR. Individual data points are shown as outliers.

**S2. Motor driver–motor interface and pin mapping**

To ensure correct integration between the motor driver and the brushless motor, the connector pin mapping was defined according to the manufacturer documentation (Maxon) and is summarized in Table S1. The power interface (J1) provides the 24 V supply required for motor operation. The motor phase connections (J2) deliver three-phase drive signals (phases A, B, and C) to the motor windings. Hall sensor outputs (J3) are used for rotor position feedback and are connected to the motor driver for commutation control. The encoder interface (J4) provides quadrature signals (A, B) and index signals, along with power and ground, enabling precise position and speed monitoring. In addition, a TTL trigger input (J5) is used to receive control signals from the external system (e.g., Open Ephys), allowing closed-loop activation of the motor. This standardized mapping ensures reliable communication between the motor and driver and facilitates reproducibility of the system setup.

Table S1 Connector pin mapping based on Maxon manufacturer documentation.

| Interface | Connector | Pin No. | Signal | Wire color | Destination |
| --- | --- | --- | --- | --- | --- |
| Power | J1 | J1-1 | GND | -- | 24V supply (-) |
| Power | J1 | J1-2 | GND | -- | 24V supply (+) |
| Motor phase | J2 | J2-1 | Motor phase A | Red | Motor lead |
| Motor phase | J2 | J2-2 | Motor phase B | Black | Motor lead |
| Motor phase | J2 | J2-3 | Motor phase C | White | Motor lead |
| Hall sensors | J3 | J3-1 | Hall sensor 1 | Yellow | Motor Hall output |
| Hall sensors | J3 | J3-2 | Hall sensor 2 | Brown | Motor Hall output |
| Hall sensors | J3 | J3-3 | Hall sensor 3 | Grey | Motor Hall output |
| Encoder | J4 | J4-1 | +5V | -- | Encoder supply |
| Encoder | J4 | J4-2 | GND | -- | Encoder ground |
| Encoder | J4 | J4-3 | A- | -- | Encoder channel A |
| Encoder | J4 | J4-4 | A+ | -- | Encoder channel A |
| Encoder | J4 | J4-5 | B- | -- | Encoder channel B |
| Encoder | J4 | J4-6 | B+ | -- | Encoder channel B |
| Encoder | J4 | J4-7 | Index- | -- | Encoder index |
| Encoder | J4 | J4-8 | Index+ | -- | Encoder index |
| TTL trigger | J5 | J4-1 | 5V TTL | -- | Motor enable signal |

**S3. Sleep state classification performance across individual mice**

To assess the consistency and robustness of the sleep state classification algorithm across subjects, we computed confusion matrices for each individual mouse. Fig. S2 presents the classification performance for all eight animals, with each panel corresponding to one mouse. Across subjects, the classifier demonstrates strong performance in distinguishing REM and NREM states, as indicated by the high diagonal values in the corresponding rows. Wake classification shows comparatively greater variability, with some degree of misclassification into NREM or REM, which is consistent with the transitional and heterogeneous nature of wakefulness. Despite inter-subject variability, the overall structure of the confusion matrices remains consistent across animals, indicating that the algorithm generalizes well without requiring subject-specific parameter tuning. These results support the robustness of the proposed method for real-time sleep state classification in freely behaving mice.

**
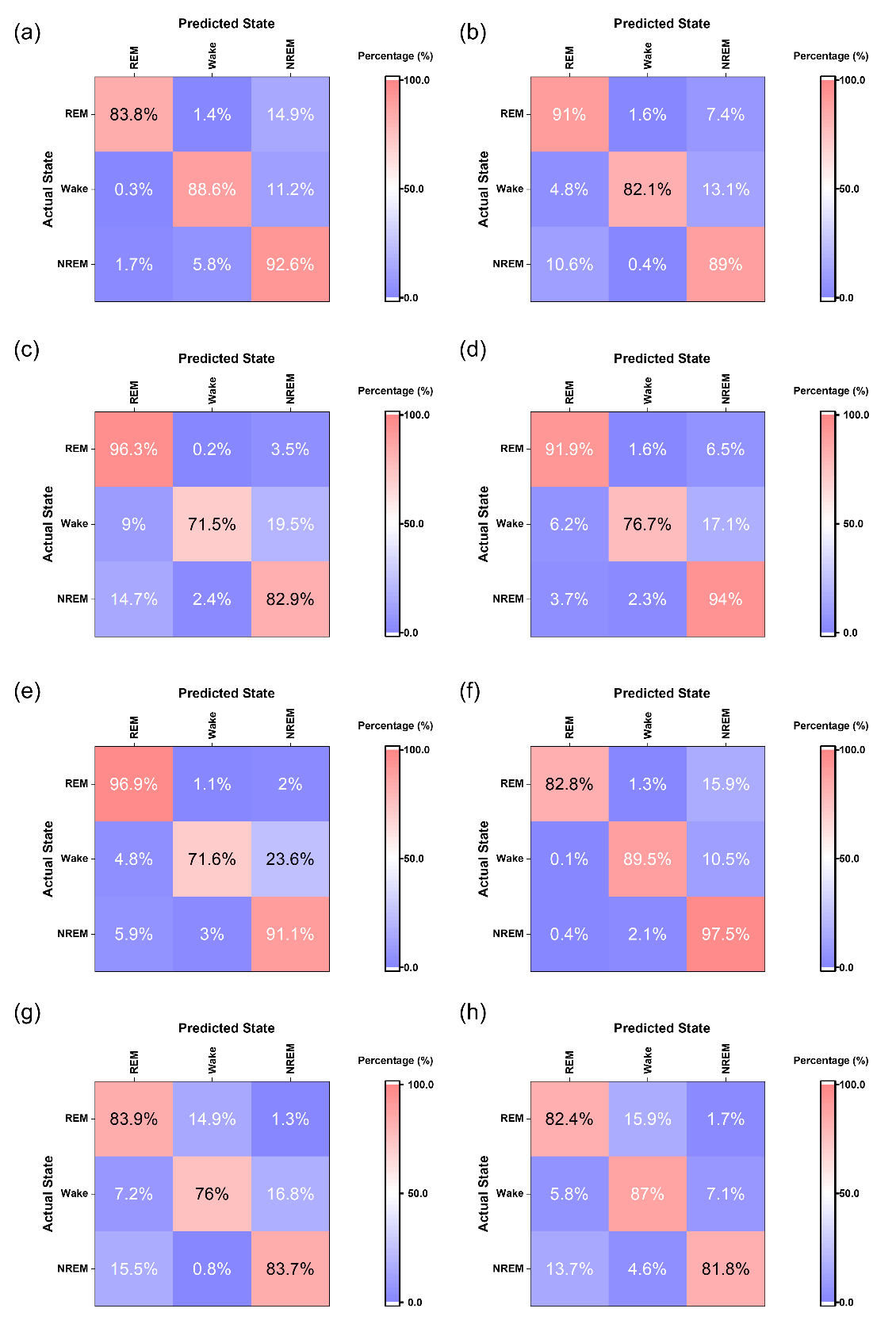
**

Fig. S2. Confusion matrices for sleep state classification across eight individual mice. Each panel ((a)–(h)) corresponds to a single mouse (Mouse #1–#8). Rows represent the true labels (actual state), and columns represent the predicted labels (REM, Wake, and NREM). Values are expressed as percentages of total epochs per true class. High values along the diagonal indicate correct classifications, whereas off-diagonal values represent misclassifications.

**S4. Quantitative classification performance across individual mice**

To quantitatively evaluate classification performance across subjects, we computed recall and F1-score for each sleep state (REM, Wake, and NREM), as well as overall accuracy for each individual mouse. The results are summarized in Table S2. Consistent with the confusion matrices shown in Fig. S2, the classifier achieves strong performance in identifying REM and NREM states across all animals, with high recall and F1-scores. In contrast, Wake classification exhibits greater variability, with relatively lower recall and F1-scores in some subjects, reflecting the more heterogeneous nature of wakefulness and its overlap with transitional states. Despite inter-subject variability, the overall classification accuracy remains consistently high across mice, indicating robust generalization of the model without subject-specific parameter tuning. These results further support the reliability of the proposed method for real-time sleep state classification. Overall, these quantitative results demonstrate stable performance across subjects and are consistent with the qualitative patterns observed in the confusion matrices。

Table S2 Classification performance for individual mice (n = 8). Values are reported as percentages.

| Mouse |  | REM Recall | Wake Recall | NREM Recall | REM F1 | Wake F1 | NREM F1 | Accuracy |
| --- | --- | --- | --- | --- | --- | --- | --- | --- |
| 1 |  | 83.76% | 88.56% | 92.56% | 90.21% | 90.51% | 84.67% | 88.29% |
| 2 |  | 91.02% | 82.07% | 89.01% | 88.17% | 89.18% | 84.98% | 87.36% |
| 3 |  | 82.78% | 89.49% | 97.47% | 90.33% | 92.81% | 87.09% | 89.92% |
| 4 |  | 83.86% | 75.96% | 83.73% | 81.20% | 79.27% | 82.98% | 81.18% |
| 5 |  | 96.32% | 71.52% | 82.89% | 87.57% | 82.16% | 80.53% | 83.58% |
| 6 |  | 91.93% | 76.68% | 94.01% | 91.11% | 84.91% | 86.42% | 87.54% |
| 7 |  | 96.91% | 71.56% | 91.13% | 93.34% | 81.49% | 81.10% | 86.53% |
| 8 |  | 82.40% | 87.04% | 81.76% | 81.62% | 83.90% | 85.81% | 83.74% |

**S5. Suppression of REM sleep under closed-loop deprivation: representative examples**

To further illustrate the effect of REM sleep deprivation at the signal level, we present representative examples of brain state classification, EEG spectrograms, and EMG signals from a single mouse under baseline and deprivation conditions (Fig. S3). During the baseline recording (no sleep deprivation), distinct REM sleep episodes are observed, characterized by sustained theta-band activity in the EEG spectrogram and reduced EMG amplitude. In contrast, under both 24 h and 48 h REM sleep deprivation conditions, REM episodes are substantially suppressed, and the recordings are dominated by wake and NREM states. The reduction in REM sleep is consistent across both deprivation durations, demonstrating the robustness of the closed-loop system. Notably, the spectral and temporal features of the EEG remain physiologically interpretable, indicating that the intervention selectively reduces REM sleep without disrupting overall signal quality. These representative examples provide qualitative confirmation of the quantitative results reported in the main text, highlighting the system’s ability to achieve sustained REM sleep suppression over multi-hour timescales. Importantly, REM suppression is achieved without eliminating NREM structure, suggesting minimal off-target disruption of overall sleep architecture.


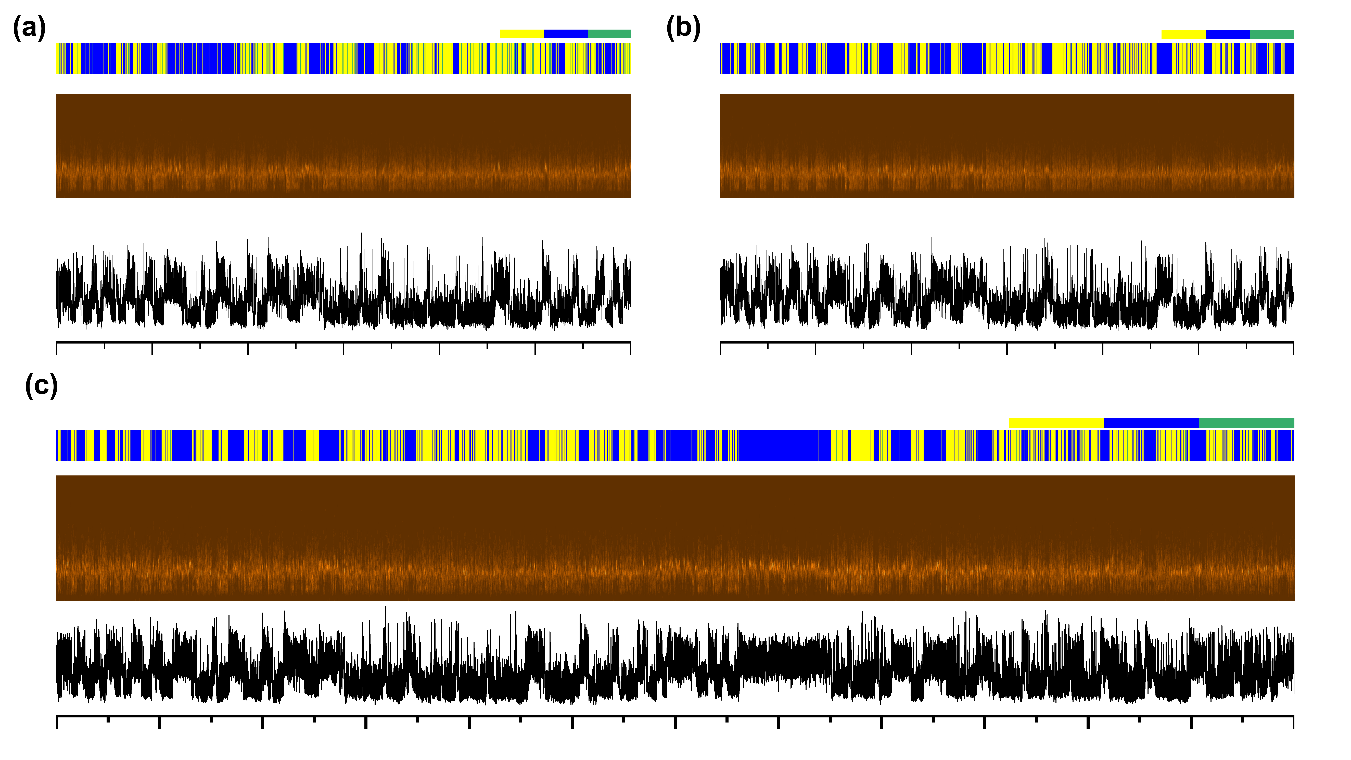


Fig. S3. Representative comparison of brain states and EEG/EMG signals under baseline and REM sleep deprivation conditions in a single mouse. (a) Baseline recording (24 h, no sleep deprivation). (b) 24 h REM sleep deprivation (SD). (c) 48 h REM sleep deprivation (SD). For each panel, the top row shows the classified brain states over time (REM, Wake, and NREM), the middle row shows the corresponding EEG spectrogram, and the bottom row shows the raw EMG signal.

**S6. Sleep state distribution across individual mice**

To quantify the effect of REM sleep deprivation across individual animals, we analyzed the distribution of sleep states (REM, Wake, and NREM) for each mouse under baseline and deprivation conditions (Table S3). Under baseline conditions, all mice exhibited measurable REM sleep, typically ranging from ~3% to 15% of total recording time. In contrast, REM sleep was consistently and substantially reduced during both 24 h and 48 h REM sleep deprivation conditions, often approaching near-zero levels. This reduction in REM sleep was accompanied by a marked increase in wakefulness, while NREM sleep showed moderate changes across animals. Importantly, the suppression of REM sleep was observed in all mice, indicating that the closed-loop system operates robustly across individuals. These results demonstrate that the REM sleep deprivation effect is not driven by a subset of animals but is consistently achieved across the cohort, further supporting the reliability and reproducibility of the system.

Table S3 Distribution of sleep states under baseline and REM sleep deprivation conditions across eight mice (n = 8). For each mouse, the percentage of time spent in REM, Wake, and NREM states is reported for 24 h baseline, 24 h and 48 h REM sleep deprivation.

|  | **24 hours Baseline** | | | **24 hours REM sleep deprivation** | | | **48 hours REM sleep deprivation** | | |
| --- | --- | --- | --- | --- | --- | --- | --- | --- | --- |
| **Mouse** | **REM** | **Wake** | **NREM** | **REM** | **Wake** | **NREM** | **REM** | **Wake** | **NREM** |
| **1** | 15.26% | 27.16% | 57.57% | 0.35% | 49.29% | 50.36% | 0.66% | 53.50% | 45.83% |
| **2** | 6.09% | 54.34% | 39.57% | 0.14% | 71.45% | 28.41% | 0.69% | 73.52% | 25.79% |
| **3** | 3.83% | 41.42% | 54.74% | 0.39% | 85.28% | 14.32% | 0.51% | 84.97% | 14.52% |
| **4** | 7.62% | 47.69% | 44.68% | 1.67% | 51.14% | 47.18% | 2.20% | 51.92% | 45.88% |
| **5** | 6.85% | 31.62% | 61.54% | 0.12% | 65.67% | 34.20% | 0.12% | 70.41% | 29.47% |
| **6** | 3.44% | 53.83% | 42.73% | 0.67% | 59.00% | 40.32% | 1.12% | 54.79% | 44.08% |
| **7** | 6.68% | 42.95% | 50.37% | 0.41% | 64.64% | 34.94% | 1.59% | 60.94% | 37.46% |
| **8** | 6.94% | 42.01% | 51.05% | 0.64% | 63.80% | 35.55% | 1.79% | 62.21% | 35.98% |
